# Antiviral Immunotoxin Against *Bovine herpesvirus*-1: Targeted Inhibition of Viral Replication and Apoptosis of Infected Cell

**DOI:** 10.3389/fmicb.2018.00653

**Published:** 2018-04-04

**Authors:** Jian Xu, Xiaoyang Li, Bo Jiang, Xiaoyu Feng, Jing Wu, Yunhong Cai, Xixi Zhang, Xiufen Huang, Joshua E. Sealy, Munir Iqbal, Yongqing Li

**Affiliations:** ^1^Beijing Key Laboratory for Prevention and Control of Infectious Diseases in Livestock and Poultry, Beijing, China; ^2^Institute of Animal Husbandry and Veterinary Medicine, Beijing Academy of Agriculture and Forestry Sciences, Beijing, China; ^3^College of Animal Science and Technology, Jiangxi Agricultural University, Nanchang, China; ^4^Beijing Center for Animal Disease Control and Prevention, Beijing, China; ^5^The Pirbright Institute, Woking, United Kingdom

**Keywords:** *Bovine herpesvirus-1*, immunotoxin, antiviral function, specific binding, targeted cytotoxic effect, apoptosis

## Abstract

*Bovine herpesvirus* 1 (BoHV-1) is a highly contagious viral pathogen which causes infectious bovine rhinotracheitis in cattle worldwide. Currently, there is no antiviral prophylactic treatment available capable of mitigating the disease impact and facilitating recovery from latent infection. In this study, we have engineered a novel recombinant anti-BoHV-1 immunotoxin construct termed “BoScFv-PE38” that consists of a single-chain monoclonal antibody fragment (scFv) fused with an active domain of *Pseudomonas* exotoxin A as a toxic effector (PE38). The recombinant BoScFv-PE38 immunotoxin expressed in a prokaryotic expression system has specific binding affinity for BoHV-1 glycoprotein D (gD) with a dissociation constant (Kd) of 12.81 nM and for BoHV-1 virus particles with a Kd value of 97.63 nM. We demonstrate that the recombinant BoScFv-PE38 is internalized into MDBK cell compartments that inhibit BoHV-1 replication with a half-maximal inhibitory concentration (IC_50_) of 4.95 ± 0.33 nM and a selective index (SI) of 456 ± 31. Furthermore, the BoScFv-PE38 exerted a cytotoxic effect through the induction of ATP and ammonia, leading to apoptosis of BoHV-1-infected cells and the inhibition of BoHV-1 replication in MDBK cells. Collectively, we show that BoScFv-PE38 can potentially be employed as a therapeutic agent for the treatment of BoHV-1 infection.

## Introduction

*Bovine herpesvirus*-1 (BoHV-1) belongs to the *Herpesviridae* family in the *Alphaherpesvirinae* subfamily (Muylkens et al., 2007) and is an economically important pathogen that causes infectious bovine rhinotracheitis (IBR) in cattle (Rola et al., 2017; Thakur et al., 2017). BoHV-1 infected animals experience a range of mild to severe clinical syndromes, including rhinotracheitis, vaginitis, balanoposthitis, abortion, conjunctivitis, and enteritis, together with reduced milk production, and weight gain (Raaperi et al., 2014). BoHV-1 pathobiology is somewhat similar to the human herpesvirus 1 (HHV-1), having a short replication cycle and the ability to cause life-long infection (Levings and Roth, 2013; Zhu et al., 2017). BoHV-1 can also serve as disease model for improving control strategies against *herpesviruses* infecting both humans and animals. Although BoHV-1 vaccines are effective at reducing the clinical impact of BoHV-1 infection, the available vaccines provide suboptimal protection against BoHV-1 in cattle (Muylkens et al., 2007). Therefore, it is necessary to develop antiviral agents that target infected cells to clear virus in host, especially act as a reservoir for spreading virus throughout a herd (Frizzo da Silva et al., 2013). Treatment of viral infections with currently available synthetic drugs possess several deficiencies including toxicity and resistance (Spiess et al., 2016; Khandelwal et al., 2017; Wambaugh et al., 2017), therefore, there is urgency for new and improved antivirals. Recently, immunotoxins against a variety of viruses have been developed, including single-stranded RNA viruses infecting humans, such as HIV, PCV, rabies virus, and herpesvirus, HCMV, EBV and HSV-2 (Mareeva et al., 2010; Chatterjee et al., 2012; Spiess et al., 2017). Immunotoxins, that are chimeric proteins consisting of the antigen-binding fragment (Fab) of an antibody conjugated to a toxin molecule, have shown promise in targeted delivery of antiviral toxins to virus infected cells (Margolis et al., 2016; Spiess et al., 2016). There is growing interest in developing immunotoxins for use in cancer treatment, and lately, the development of a variety of immunotoxins has been reported with the ability to inhibit virus replication and dissemination along with destruction and clearance of infected cells (Mazor et al., 2012; Denton et al., 2014; Chandramohan et al., 2017; Lim et al., 2017; Polito et al., 2017). The major beneficial effect of antibody-conjugated immunotoxins is that they are selective and provide targeted delivery of toxins with minimal side effects to the host (Cai and Berger, 2011; Hou et al., 2016; Müller et al., 2017). Therefore, the target molecule is the major element within the immunotoxin and plays a vital role in targeting virus-infected cells.

The targeting of cell surface antigens or pathogens is usually achieved through the use of their specific monoclonal antibodies (mAbs). The Fab portion of mAbs can be genetically engineered as a recombinant single-/double-chain antibody fragment, or constructed as a single-chain antibody fragment (scFv) for use a as a targeting molecule. These scFv molecules have been used in various immunotoxins due to its high specificity and binding ability. Furthermore, scFv displays good biocompatibility with low antigenicity and may not elicit an immune response when administered to animals and humans (Schotte et al., 2014; Della Cristina et al., 2015; Hanke et al., 2016; Liu B. et al., 2016). Bacterial toxins (*Pseudomonas* exotoxin or *diphtheria* toxin) are most commonly used to prepare immunotoxins, due to irreversibly inhibit protein synthesis in eukaryotic cells via ADP-ribosylation of translation elongation factor 2 (eEF2) (Chatterjee et al., 2012; Spiess et al., 2016).

In our previous study, we demonstrated that scFv targeting of viral glycoprotein D (gD) inhibited the infectivity of BoHV-1 in Madin-Darby bovine kidney (MDBK) cells (Xu et al., 2017). In the present study, we developed BoHV-1-specific scFv that acted as the targeting molecule. Recombinant bacterial toxin derived from *Pseudomonas* exotoxin A (PE38) linked with BoHV-1-specific scFv (BoScFv-PE38) showed immunotoxin activity by binding to BoHV-1 particles in virus-infected cells and exerting a specific cytotoxic effect through induction of high levels of ATP and ammonia production, leading to apoptosis of BoHV-1-infected cells. As a result, replication of BoHV-1 was significantly reduced in MDBK cells.

## Materials and Methods

### Cells and Viruses

MDBK cells and human embryonic kidney HEK293T (293T) cells were purchased from the American Type Culture Collection (Manassas, VA, United States). The MDBK and 293T cell lines were cultured at 37°C in a 5% CO_2_ incubator in Dulbecco’s modified Eagle’s medium (DMEM; Invitrogen) supplemented with 10% fetal bovine serum. *Bovine herpesvirus* 1 (BoHV-1) (BK1952) was obtained from the China Veterinary Culture Collection Center (CVCC), Beijing, China, and grown in MDBK cells.

### Plasmids and Antibodies

The pET28a expression system was obtained from GE Healthcare; the pEGFP-N1 vector was obtained from Clontech; The DNA encoding for segment from 259 amino acids to 345 amino acids of glycoprotein D (AFB76672.1) was amplified by polymerase chain reaction with the primers as following: Sense primer: 5′-GAATTCATGGAGGAGTCGAAGGGC-3′ and anti-sense primer:5′-CTCGAGGATGGCTTCGAGGCTCG-3′, and the DNA fragment was cloning into the pEGFP-N1 vector for construction of the pEGFP-N1-gD, which was used to efficiently express green fluorescent protein (GFP) fused gD protein in 293T cell. Calf antiserum against BoHV-1 was from China Veterinary Culture Collection Center. A mouse anti-His monoclonal antibody (McAb), Alexa Fluor 555- conjugated anti-His antibody, FITC-labeled goat anti-mouse antibody and TRITC-labeled goat anti-mouse antibody were purchased from ThermoFisher Scientific (United States), and a FITC-labeled rabbit anti-bovine antibody was purchased from BioVision (United States). The antibodies against PARP-1, Bcl-2, Bid, caspase-3, caspase-8, caspase-9 and β-actin were purchased from ABclonal Biotech (China). The BoScFv-PE38 was labeled with horseradish peroxidase (HRP) by Sangon Biotech Co., Ltd. (China).

### Sequence Analysis and Expression of the Immunotoxin

The protein sequence of *Pseudomonas* exotoxin A (PE38) was downloaded from the NCBI database^1^; sequences of a truncated version of PE38, the BoHV-1 ScFv protein and the linker peptide are obtained according to our earlier study (Xu et al., 2017), which were listed in Supplementary Table S1. The domains of BoScFv-PE38 were analyzed using the PROSITE database^2^. The full-length nucleotide sequence of BoScFv-PE38 was optimized and synthesized by Shanghai Sangon Biotech Co., Ltd. (Shanghai, China). The fusion gene BoScFv-PE38 was cloned into expression vector pET28a and expressed in *Escherichia coli* BL21 (DE3) (Novagen, EMD Chemicals, Inc., Madison, WI, United States). The recombinant His tagged BoScFv-PE38 was purified via nickel affinity chromatography as described by Della Cristina et al. (2015). The endotoxin was removed from purified BoScFv-PE38 using the Detoxi-Gel^™^ Endotoxin Removing Columns Kit (ThermoFisher Scientific, United States), and the endotoxin residue in purified BoScFv-PE38 was detected with the ToxinSensor^™^ Chromogenic LAL Endotoxin Assay Kit (Kingsy Biotechnology, Nanjing, China). The purified BoScFv-PE38 (endotoxin < 0.0068 EU/ml) was dissolved in phosphate-buffered saline (PBS, pH 7.4) solution and stored at -20°C.

### Measurement of the Dissociation Constants of BoScFv-PE38

The Kd of BoScFv-PE38 was measured via ELISA. Briefly, 96-well microplates were coated with the BoHV-1 or gD protein (expressed and purified from *E. coli*) and blocked with 3% bovine serum albumin (BSA). Serial dilutions of BoScFv-PE38 labeled with horseradish peroxidase (HRP) (HRP-BoScFv-PE38) (0–100 nM for the gD protein, 0–1500 nM for BoHV-1) were added to the wells, followed by incubation at 37°C for 60 min. Next, the tetramethylbenzidine (TMB) (Sigma) substrate was added, followed by incubation for 10 min. Then, stop buffer (2 M sulfuric acid) was added to stop the reaction. Finally, the optical densities were read at 450 nm, and the equation Y = Bmax X/(Kd+X) was used to obtain the saturation curve and Kd of BoScFv-PE38, employing GraphPad Prism 5.0. Y represents the mean OD450 nm value; Bmax is the maximal OD450 nm value; and X is the concentration of BoScFv-PE38.

### Immunofluorescence Assay and Confocal Laser Scanning Microscopy

The Immunofluorescence assay (IFA) was performed as described previously (Keuser et al., 2004). Briefly, 293T cells were seeded onto cover slips in six-well plates and cultured to 70% confluency at 37°C over 18–24 h. Then, the PEGF-N1 and PEGF-N1-gD plasmids were transfected with Lipofectamine 3000 (Life Technology, United States) according to the manufacturer’s instructions. After 6 h, BoScFv-PE38 (1 μM) was added to the culture medium, and the 293T cells were cultured for an additional 24 h. Then, the 293T cells were fixed with 4% paraformaldehyde, blocked with 3% bovine serum albumin, and incubated with the Alexa Fluor 555-anti-His antibody at 37°C for 1 h. Finally, the nuclei were counterstained with DAPI (blue), and the cell samples were examined under a fluorescence microscope (Leica EL 6000). The co-localization of gD with BoScFv-PE38 or the karyomorphism in 293T cells was observed under a confocal laser scanning microscopy (CLSM, Leica).

MDBK cells were seeded on cover slips in six-well plates and cultured to 50% confluency at 37°C for 18–24 h. Then, the MDBK cells were infected with BoHV-1 (MOI = 1). After 1.5 h, the culture medium was replaced with fresh culture medium containing 1% FBS and BoScFv-PE38 (1 μM), and the MDBK cells were cultured for an additional 24 h. Next, the MDBK cells were fixed with 4% paraformaldehyde and blocked with 3% bovine serum albumin. To detect the binding of BoScFv-PE38 in BoHV-1-infected cells, the cells were incubated with the anti-His McAb at 37°C for 1 h, followed by incubation with the FITC-labeled goat anti-mouse antibody at 37°C for 1 h. Sequently, in order to monitor the localization of BoHV-1 or BoHV-1 gD with BoScFv-PE38, the cells were incubated with the anti-gD McAb or anti-BoHV-1 Bovine serum at 37°C for 1 h. After washing three times with PBS, the cells were incubated with the FITC-labeled goat anti-mouse antibody or FITC-labeled rabbit anti-bovine antibody, respectively, at 37°C for 1 h. The cells were next washed three times with PBS and subsequently incubated with Alexa Fluor 555-conjugated anti-His antibody at 37°C for 1 h. Finally, the nuclei were counterstained with DAPI (blue), and cell samples were examined with a fluorescence microscope (Leica EL 6000). The co-localization of gD and BoScFv-PE38 and the karyomorphism of MDBK cells were observed under a CLSM (Leica).

### Cytotoxicity of BoScFv-PE38 to 293T Cells Expressing gD

293T cells were seeded in 96-well plates and cultured to 70% confluency at 37°C for 18–24 h. Then, the PEGF-N1 and PEGF-N1-gD plasmids were transfected with Lipofectamine 3000 (Life Technology, United States) according to the manufacturer’s instructions. After 6 h, BoScFv-PE38 (0–1.0 μM) was added to the culture medium, followed by cultivation at 37°C for 24 h. Cell proliferation was tested with the CellTiter 96 Aqueous One Solution Cell Proliferation Assay (MTS) Kit (Promega) according to the manufacturer’s instructions, and the OD490 values of the test wells were read with a microplate reader, the cellular viability was calculated according to the equation: Cellular viability (%) = OD490 value_(Treatment group)_/OD490 value _(Control group)_× 100. All tests were performed in triplicate.

### Cytotoxicity of BoScFv-PE38 to MDBK Cells Infected With BoHV-1

To determine a proper dose of BoHV-1 for inoculating MDBK cells, the cells were seeded in 96-well plates and cultured to 90% confluency. Then, the MDBK cells were inoculated with a 0.01–10 multiplicity of infection (MOI) of BoHV-1 at 37°C for 1.5 h. Then, the culture medium was replaced with fresh culture medium containing 1% FBS and BoScFv-PE38 (1.00 μM), and the cells were cultured for 24 h at 37°C. Cellular viability in each plate was calculated via the MTS assay in quadruplicate. Finally, the specific cytotoxicity of BoScFv-PE38 was also evaluated using the MTS assay as described above with 1 MOI of BoHV-1 and 0–1 μM BoScFv-PE38. Each experiment was repeated four times.

### Analysis of Apoptosis

Titration of ATP and ammonia in MDBK cells was performed as follows. MDBK cells were seeded on cover slips in 96-well plates and cultured to 90% confluency at 37°C for 18–24 h. The MDBK cells were then infected with BoHV-1 (MOI = 1) and cultivated at 37°C for a further 1.5 h. Then, the culture medium was replaced with fresh culture medium containing 1% FBS and BoScFv-PE38 (0.015625, 0.03125, 0.0625, 0.125, 0.25, 0.50, 1.00, and 2.00 μM), and the cells were cultured for an additional 24 h. The concentrations of ATP and ammonia were measured with an ATP determination kit (Sigma) and an ammonia assay kit (Sigma) according to the manufacturer’s instructions.

The TUNEL assay was employed as described previously (Hohensinner et al., 2017). MDBK cells were seeded in 24-well plates and cultured to 90% confluency at 37°C over 18–24 h. Then, the MDBK cells were infected with BoHV-1 (MOI = 1) and cultivated at 37°C for 1.5 h, followed by culture in medium containing 1% FBS and BoScFv-PE38 (0.125, 0.25, 0.5, and 1 μM) for 24 h. Apoptosis-positive MDBK cells were detected with the *In Situ* Cell Death Detection Kit, AP (Roche) according to the manufacturer’s instructions, and the apoptotic rates of MDBK cells in the presence of different concentration of BoScFv-PE38 were determined.

### Western Blot Analysis

The purified BoHV-1 gD protein (20 μg) or BoHV-1 (20 μg) was separated through sodium dodecyl sulfate–polyacrylamide gel electrophoresis (SDS-PAGE) and then transferred to a polyvinylidene difluoride (PVDF) membrane (Millipore Schwalbach, Germany). Next, the membrane was blocked with 5% bovine skimmed milk and then incubated with BoScFv-PE38 labeled with horseradish peroxidase (HRP) at 37°C for 1.5 h. Thereafter, the membrane was washed three times with PBS containing 0.5% Tween-20. The membrane was finally developed with ECL solution (ThermoFisher Scientific, United States) and visualized with the Odyssey Infrared Imaging System (LI-COR Biosciences).

The expression of apoptotic proteins in BoHV-1-infected MDBK cells after treatment with BoScFv-PE38 was analyzed as described by Decker et al. (2004). Fifty micrograms of protein was separated via 12% SDS-PAGE and transferred to PVDF membranes. The samples were subsequently hybridized with antibodies against PARP-1, Bcl-2, Bid, caspase-3, caspase-8, caspase-9 and β-actin (ABclonal Biotech Co., Ltd, China). Blots were developed using Super Signal chemoluminescent substrates (Pierce, KMF GmbH, St. Augustin, Germany).

### Plaque Reduction Assay

The antiviral activity of BoScFv-PE38 was further evaluated through the plaque reduction test (PRT) as previously described (Levings et al., 2015). BoScFv-PE38 (0.25, 0.5, 1, and 2 μM) was mixed with 10–50% tissue culture infective dose (TCID_50_) of BoHV-1 in an equal volume. After incubation at 37°C under 5% CO_2_ for 1 h, 1 ml of the mixture was inoculated in triplicate into the wells of a six-well plate containing a confluent monolayer of MDBK cells. The plates were subsequently incubated at 37°C under 5% CO_2_ for 1.5 h with intermittent rocking. Then, an agarose overlay was added to the infected cell monolayer. After the agarose became solid, the plates were placed upside down and were further incubated for 48 h. When viral plaques became visible, the cells were fixed with 4% formaldehyde and stained with 0.1% toluidine blue in saline, followed by visual counting of viral plaques.

### Infectious Center Assay

The infectious center assay (ICA) was employed as reported previously (Geoghegan et al., 2015). MDBK cells were handled, as described above, and treated with BoScFv-PE38 (0.125, 0.25, 0.5, and 1 μM) for 24 h. All cultures were harvested and subjected to repeated freeze-thaw three times at 2, 10, 16, and 24 h. The culture mixture (harvested at 2 h) was diluted to 1:100–1:1000. The diluted culture mixtures were used to infect MDBK cells in 6-well plates for 1.5 h. An agarose overlay was then added to the infected cell monolayer, and after the agarose solidified, the plates were placed upside down and further incubated for 48 h, and viral plaques were counted visually.

### 50% Cytotoxic Concentration, 50% Inhibitory Concentration and Selective Index

To evaluate the cytotoxic concentration of BoScFv-PE38, MDBK cells were seeded in 96-well plates and cultured to 90% confluency at 37°C for 18–24 h. Then, BoScFv-PE38 (0.1263–20.0 μM) was added to the culture medium, followed by cultivation at 37°C for 24 h. Cell proliferation was tested with the CellTiter 96 Aqueous One Solution Cell Proliferation Assay (MTS) Kit (Promega) according to the manufacturer’s instructions, and the OD490 values of the test wells were read with a microplate reader for calculation of the cellular viability, and the cytotoxic concentration and 50% cytotoxic concentration (CC_50_) was calculated. To determine the 50% inhibitory concentration (IC_50_) of BoScFv-PE38, twofold serial dilutions of BoScFv-PE38 (initial 250 nM) were incubated with BoHV-1 (MOI = 1) for 1 h at 37°C. Thereafter, the mixtures were added to MDBK cells, followed by culture for 72–96 h, and the cytopathic effect was observed and calculated as the IC_50_ value. The selective index (SI) was calculated according to the equation: SI = CC_50_/IC_50_. All tests were performed in triplicate (Supplementary Table S2).

### Statistics

All the statistical analyses were performed via analysis of variance (ANOVA) using SPSS software, version 18.0, and the fitting curves were drawn with GraphPad Prism 5.0. *P*-values < 0.01 were considered statistically significant. *P* > 0.05 represents no statistically significant differences. *P* < 0.05 represents statistically significant differences. *P* < 0.01 represents statistically significant differences. *P* < 0.001 represents statistically significant differences.

## Results

### Construction of BoScFv-PE38 Immunotoxin

**FIGURE 1:**
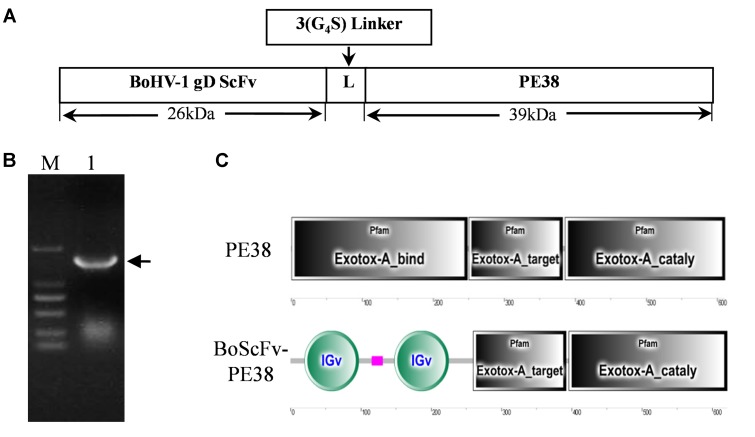
Nucleotide sequence structure of BoScFv-PE38. **(A)** Schematic diagram of the recombinant immunotoxin BoScFv-PE38. BoHV-1 gD scFv, BoHV-1-specific nanobody; 3(G_4_S) linker, flexible linkers consisting of glycine and serine residues; PE38, pseudomonas exotoxin A (PE38) protein without the Exotox-A-binding region. **(B)** The BoScFv-PE38 nucleotide sequence. M represents the DNA molecular weight marker; 1 represents the BoScFv-PE38 nucleotide sequence; and the arrow indicates the size of the BoScFv-PE38 nucleotide sequence. **(C)** Structural sequence analysis of BoScFv-PE38 and PE38.

A DNA fragment containing the open reading frame of PE38 was synthesized according to the published sequence of *Pseudomonas* exotoxin A (PE38), derived from *Pseudomonas aeruginosa*, without an intrinsic cell-binding domain (**Figure 1A**). The modified PE38 nucleotide sequence was then fused with a gene encoding a scFv mAb that has high specificity toward the gD protein of BoHV-1; the result was a 1863 bp construct of BoScFv-PE38 immunotoxin (**Figure 1B** and Supplementary Table S1). In the BoScFv-PE38 construct, the Exotox-A-binding region of PE38 was replaced with an scFv against the gD protein of BoHV-1, but the Exotox-A-targeting and Exotox-A catalytic domains were maintained and ligated with the binding domain of scFv via a 3 (G_4_S) linker (**Figure 1C**).

### BoScFv-PE38 Immunotoxin Showed Specific Binding Affinity for BoHV-1

**FIGURE 2:**
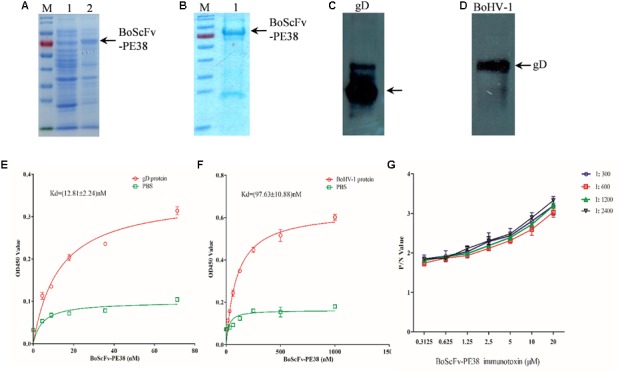
Expression and binding analysis of BoScFv-PE38. **(A)** Expression of BoScFv-PE38 in *E. coli*. M: PageRuler Prestained Protein Ladder; 1: the pET28a vector was transformed into *E. coli* cells, and expression was induced with IPTG; 2: the pET28a- BoScFv-PE38 vector was transformed into *E. coli* cells, and expression was induced with IPTG. The arrow represents the recombinant ScFv. **(B)** Purification of BoScFv-PE38 from *E. coli* via nickel affinity chromatography. M: PageRuler Prestained Protein Ladder. 1 indicates purified BoScFv-PE38, and the arrow represents purified ScFv. **(C)** The reaction between gD and BoScFv-PE38 was detected by western blotting. Recombinant gD protein detected by BoScFv-PE38-HRP. **(D)** The reaction between BoHV-1 and BoScFv-PE38 was detected by western blotting. BoHV-1: BoHV-1 was detected by BoScFv-PE38-HRP. **(E)** Measurement of the Kd of BoScFv-PE38 binding to the gD protein. First, 96-well plates were coated with gD (1 μg). The plates were then incubated with BoScFv-PE38-HRP and developed with TMB substrate solution. The reaction was stopped with 2 M sulfuric acid, and the optical density was read at 450 nm. The equation Y = BmaxX/(Kd + X) was used to obtain the saturation curve and the Kd of the ScFv-gD interaction using GraphPad Prism 5.0. Y represents the mean OD450 value; Bmax is the maximal OD450 value; and X is the concentration of BoScFv-PE38. **(F)** Measurement of the Kd of BoScFv-PE38 binding to BoHV-1. Ninety-six-well plates were coated with BoHV-1 (1 μg), and the Kd of the BoScFv-PE38-BoHV-1 interaction was measured as described above. **(G)** Binding affinity of BoScFvScFv-PE38 was detected using an antigen capture ELISA. Ninety-six-well microplates were coated with different concentrations of BoScFvScFv-PE38 and blocked with 3% BSA. The BoHV-1 antigen (standard serially diluted BoHV-1-positive serum; 1:300–1:2400), and HRP-conjugated goat anti-bovine IgG were added, and the plates were developed with the TMB substrate (Sigma). The reaction was stopped with 2 M sulfuric acid, and the optical density was read at 450 nm. The P/N values were analyzed using GraphPad Prism 5.0.

To assess the binding affinity of BoScFv-PE38 immunotoxin to the BoHV-1, the DNA fragment of BoScFv-PE38 was cloned into the pET28a plasmid and expressed in *Escherichia coli* BL21(ED3) (**Figure 2A**). The BoScFv-PE38 immunotoxin was then purified via affinity chromatography (**Figure 2B**). The western blot analysis showed that both the recombinant gD protein produced in *E. coli*. and the wild type gD protein derived from BoHV-1 virus had specific binding affinity for purified BoScFv-PE38 immunotoxin (**Figures 2C,D**). Enzyme-linked immunosorbent assay (ELISA) analysis also showed very high binding avidity of BoScFv-PE38 immunotoxin with the recombinant gD protein and BoHV-1 (**Figures 2C,D**). The dissociation constants (Kds) of BoScFv-PE38 binding to gD and BoHV-1 were 12.81 ± 2.24 and 97.63 ± 10.88, respectively (**Figures 2E,F**), indicating that the BoScFv-PE38 protein had specific and high binding avidity for both the BoHV-1 gD and BoHV-1 virus particles. To further test the ability of BoScFv-PE38 immunotoxin to capture BoHV-1 virus, a double sandwich ELISA was established by coating a 96-well microplate with different doses of BoScFv-PE38. Then, the amount of BoHV-1 antigen that was captured was titrated via ELISA. As shown in **Figure 2G**, there was a positive correlation between the amounts of BoHV-1 antigen captured and the concentrations of BoScFv-PE38 immunotoxin. The comparative control assays showed no significant differences among the tested dilutions (1:300–1:2400) of standard bovine serum against BoHV-1 (**Figure 2G**).

### BoScFv-PE38 Immunotoxin Interacts With BoHV-1 gD Protein Within Cells

**FIGURE 3:**
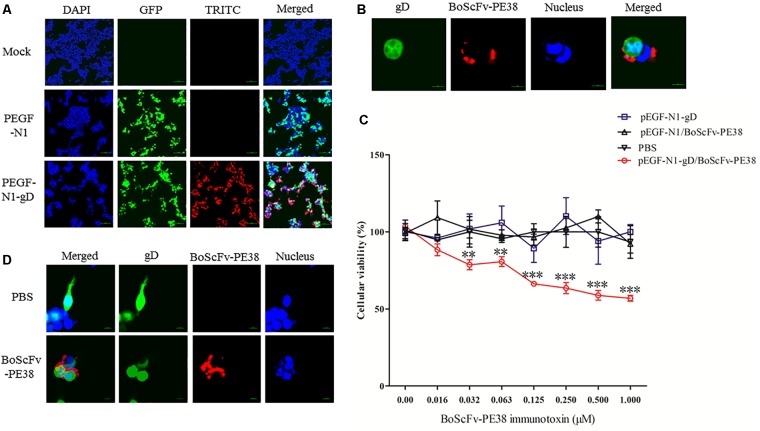
Binding specificity and cytotoxicity of BoScFv-PE38 in gD-expressing 293T cells. **(A)** Binding specificity of BoScFv-PE38 in gD-expressing 293T cells determined via IFA. 293T cells were seeded on cover slips in six-well plates and cultured to 70% confluency at 37°C over 18–24 h. Then, the PEGF-N1 and PEGF-N1-gD plasmids were transfected into the cells. After 6 h, BoScFv-PE38 (1 μM) was added to the culture medium, and the 293T cells were cultured for an additional 24 h. The 293T cells were subsequently fixed with 4% paraformaldehyde, permeabilized with 0.1% Triton X-100 for 1 h, blocked with 3% BSA, and incubated with the Alexa Fluor 555-anti-His antibody at 37°C for 1 h. Finally, the nuclei were counterstained with DAPI (blue), and the cell samples were examined with a fluorescence microscope (Leica EL 6000). **(B)** Localization of BoScFv-PE38 in gD-expressing 293T cells determined via CLSM. The 293T cells were treated as described above. Then, 293T cell samples were examined with a confocal laser-scanning microscope (Leica). **(C)** Cytotoxicity of BoScFv-PE38 in gD-expressing 293T cells. 293T cells were seeded on cover slips in 96-well plates and cultured to 70% confluency at 37°C over 18–24 h. Then, the PEGF-N1 and PEGF-N1-gD plasmids were transfected with Lipofectamine 3000 (Life Technology, United States) according to the manufacturer’s instructions. After 6 h, BoScFv-PE38 (0–1.0 μM) was added to the culture medium, followed by cultivation at 37°C for 24 h. Then, cell proliferation activity was measured with the CellTiter 96 Aqueous One Solution Cell Proliferation Assay (MTS) Kit (Promega) according to the manufacturer’s instructions, and the OD490 values of the test wells were read with a microplate reader for calculation of the cellular viability. All the statistics were analyzed through analysis of variance (ANOVA) using SPSS software, version 18.0, and the fitting curves of various concentrations of BoScFv-PE38 were drawn with GraphPad Prism 5.0, respectively. “^∗∗^” represents statistically significant differences (*P* < 0.01). “^∗∗∗^” represents statistically very significant differences (*P* < 0. 001). **(D)** Karyomorphism of gD-expressing 293T cells after treatment with BoScFv-PE38. 293T cells were seeded on cover slips in six-well plates and cultured to 70% confluency at 37°C over 18–24 h. Then, the PEGF-N1-gD plasmid was transfected into the cells. After 6 h, BoScFv-PE38 (1 μM) was added to the culture medium (PBS treated, as the negative control), and the 293T cells were cultured for 24 h. The 293T cells were then fixed with 4% paraformaldehyde, permeabilized with 0.1% Triton X-100 for 1 h, blocked with 3% BSA, and incubated with the Alexa Fluor 555-anti-His antibody at 37°C for 1 h. The nuclei were counterstained with DAPI (blue). Then, the karyomorphism of 293T cell samples was examined with a confocal laser-scanning microscope (Leica).

To determine whether BoScFv-PE38 immunotoxin could specifically target gD protein of BoHV-1 within the cellular organelles, the pEGF-N1-gD construct was engineered and transfected into 293T cells (Supplementary Figure S1). Then, 293T cells expressing the BoHV-1 gD protein were detected by staining with BoScFv-PE38 and an Alexa Fluor 555-conjugated mouse anti-His antibody. Indirect immunofluorescence assays (IFAs) and confocal microscopic assays confirmed that both the gD protein (labeled for red fluorescence) and the BoScFv-PE38 immunotoxin (labeled for green fluorescent protein (GFP)) were co-localized inside the cells (**Figures 3A,B**).

### BoScFv-PE38 Immunotoxin Has Specific Cytotoxic Activity for Cells Expressing BoHV-1 gD Protein

The specific cytotoxic activity of BoScFv-PE38 immunotoxin for cells expressing BoHV-1 gD protein was evaluated using a 3-(4,5-dimethylthiazol-2-yl)-5-(3-carboxyme-thoxyphenyl)-2- (4-sulfophenyl)-2H-tetrazolium (MTS) assay. The protein BoScFv-PE38 was added to 293T cells expressing either GFP-fused gD or GFP, followed by co-culture for 18 h. The results showed that BoScFv-PE38 could significantly inhibit the proliferation of 293T cells expressing BoHV-1 gD protein in a dose-dependent manner, but there was no obvious cytotoxic effect on control GFP-expressing 293T cells (**Figure 3C**). These results were supported by the observation of cell karyomorphism, in that there was an abnormal morphology of BoScFv-PE38-treated gD-expressing 293T cells compared with PBS-treated gD-expressing 293T cells (**Figure 3D**).

### BoScFv-PE38 Immunotoxin Captures BoHV-1 in MDBK Cells

**FIGURE 4:**
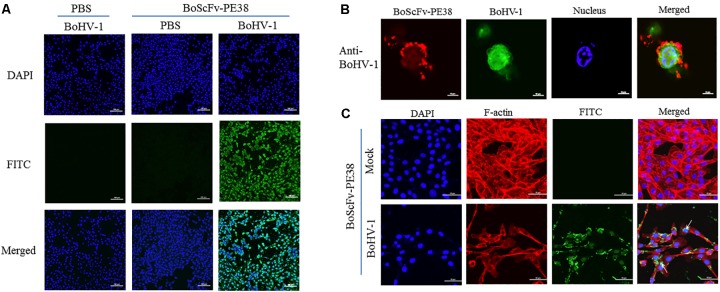
Binding specificity and internalization of BoScFv-PE38 in BoHV-1-infected MDBK cells. **(A)** Recognition of BoScFv-PE38 to BoHV-1 infected in MDBK cells was determined via IFA. MDBK cells were seeded on cover slips in six-well plates and cultured to 50% confluency at 37°C over 18–24 h. Then, the MDBK cells were infected with BoHV-1 (MOI = 1). After 1.5 h, the culture medium was replaced by new culture medium containing 1% FBS and BoScFv-PE38 (1 μM), and the MDBK cells were cultured for 24 h. The MDBK cells were fixed with 4% paraformaldehyde, permeabilized with 0.1% Triton X-100 for 1 h, blocked with 3% bovine serum albumin, incubated with the anti-His McAb for 1 h at 37°C, and then incubated with the FITC-goat anti-mouse antibody for 1 h at 37°C. The nuclei were counterstained with DAPI (blue). The cell samples were examined with a fluorescence microscope (Leica EL 6000). **(B)** Location of BoScFv-PE38 in BoHV-1-infected MDBK cells was determined via CLSM. MDBK cells were seeded on cover slips in six-well plates and cultured to 50% confluency at 37°C over 18–24 h. Then, the MDBK cells were infected with BoHV-1 (MOI = 1). After 1.5 h, the culture medium was replaced with new culture medium containing 1% FBS and BoScFv-PE38 (1 μM), and the MDBK cells were cultured for a further 24 h. The MDBK cells were then fixed with 4% paraformaldehyde, permeabilized with 0.1% Triton X-100 for 1 h, blocked with 3% bovine serum albumin, incubated with the anti-gD mAb or anti-BoHV-1 bovine serum for 1 h at 37°C, washed three times with PBS, incubated with the FITC-labeled goat anti-mouse antibody or the FITC-labeled rabbit anti-bovine antibody at 37°C for 1 h, washed three times with PBS once more, and finally incubated with the Alexa Fluor 555- anti-His antibody at 37°C for 1 h. The nuclei were counterstained with DAPI (blue). The cell samples were observed under a confocal laser-scanning microscope (Leica). **(C)** The internalization of BoScFv-PE38 entrapped by BoHV-1. MDBK cells were infected with BoHV-1 (MOI = 1). After 1.5 h, the culture medium was replaced with new culture medium containing 1% FBS and BoScFv-PE38 (1 μM), and the MDBK cells were cultured for 24 h. MDBK cells were fixed with 4% paraformaldehyde, permeabilized with 0.1% Triton X-100 for 1 h, and blocked with 3% bovine serum albumin. The cells were then incubated with the anti-His mAb for 1 h at 37°C, washed three times with PBS, and incubated with the FITC-labeled goat anti-mouse antibody at 37°C for 1 h. Cellular F-actin was stained with Alexa Fluor 555-conjugated phalloidin. The nuclei were counterstained with DAPI (blue). Normal MDBK cells were used as the negative control. Cell samples were examined with a confocal laser-scanning microscope (Leica). The white arrows indicate BoScFv-PE38 in the nucleus and cytoplasm of BoHV-1-infected MDBK cells.

The ability of BoScFv-PE38 immunotoxin to specifically recognize and interact with BoHV-1 virus particles within infected cells was investigated through IFA. The results showed that BoScFv-PE38 immunotoxin selectively internalized into the MDBK cells infected with BoHV-1 (green fluorescence) rather than negative control cells (**Figure 4A**), indicating that BoScFv-PE38 immunotoxin had efficient capability to target and internalize BoHV-1 in MDBK cells. To verify this, we observed the distribution of BoHV-1 virus particles bound to BoScFv-PE38 immunotoxin or BoHV-1 virus-specific polyclonal antibodies within an individual cell using a confocal laser-scanning microscope (CLSM). The results indicated that BoHV-1 virions recognized by BoScFv-PE38 immunotoxin were localized in the cytoplasm and to the periphery of the nucleus. In contrast, polyclonal antibodies were only able to detect virus presence at the cell surface (**Figure 4B**). These analyses also revealed that the BoScFv-PE38 immunotoxin was widely distributed on the cell surface as well as in cytoplasm of BoHV-1 infected MBCK cells (**Figure 4C**). Taken together, these results suggest that BoScFv-PE38 immunotoxin is readily internalized into the BoHV-1 infected cells following binding to the BoHV-1 gD protein expressed on the cell surface.

### BoScFv-PE38 Immunotoxin Displays Specific Cytotoxic Effect for BoHV-1-Infected MDBK Cells

**FIGURE 5:**
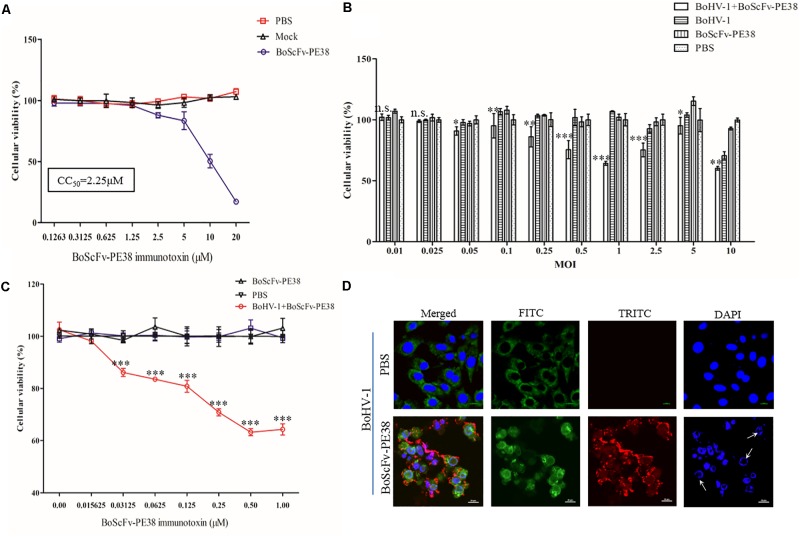
Cytotoxicity of BoScFv-PE38 in BoHV-1-infected MDBK cells. **(A)** Determination of the cytotoxic concentration of BoScFv-PE38 in MDBK cells. MDBK cells were seeded in 96-well plates and cultured to 90% confluency at 37°C for 18–24 h. Then, BoScFv-PE38 (0.1263–20.0 μM) was add to the culture medium, followed by cultivation at 37°C for 24 h, and the cellular viability was measured. **(B)** Cytotoxicity of BoScFv-PE38 in MDBK cells infected with different MOIs of BoHV-1. MDBK cells were seeded in 96-well plates and cultured to 90% confluency at 37°C over 18–24 h. Then, the MDBK cells were infected with BoHV-1 (MOI was from 0.01 to 10), added to the culture medium, and cultivated 37°C for 1.5 h. The culture medium was subsequently replaced with new culture medium containing 1% FBS and BoScFv-PE38 (1 μM); the MDBK cells were cultured for an additional 24 h; and cellular viability was measured. **(C)** Dose-dependent cytotoxicity of BoScFv-PE38 in BoHV-1-infected MDBK cells. MDBK cells were seeded on cover slips in 96-well plates and cultured to 90% confluency at 37°C for 18–24 h. Then, the MDBK cells were infected with BoHV-1 (MOI = 1), added to the culture medium, and cultivated at 37°C for 1.5 h. The culture medium was subsequently replaced with new culture medium containing 1% FBS and BoScFv-PE38 (1 μM); the MDBK cells were cultured for 24 h; and cellular viability was measured. All statistical analyses were performed via analysis of variance (ANOVA) using SPSS software, version 18.0, and the fitting curves of various concentrations of BoScFv-PE38 were drawn with GraphPad Prism 5.0. “n.s.” represents no statistically significant differences (*P* > 0.05). “^∗^” represents statistically significant differences (*P* < 0.05). “^∗∗^” represents statistically significant differences (*P* < 0.01). “^∗∗∗^” represents statistically significant differences (P < 0.001). **(D)** Karyomorphism of BoHV-1-infected MDBK cells after treatment with BoScFv-PE38. MDBK cells were seeded on cover slips in six-well plates and cultured to 50% confluency at 37°C for 18–24 h. Then, the MDBK cells were infected with BoHV-1(MOI = 1). After 1.5 h, the culture medium was replaced with new culture medium containing 1% FBS and BoScFv-PE38 (1 μM) or PBS as the negative control, and the MDBK cells were cultured for 24 h. The MDBK cells were subsequently fixed with 4% paraformaldehyde, permeabilized with 0.1% Triton X-100 for 1 h, and blocked with 3% bovine serum albumin. The cells were then incubated with anti-BoHV-1 bovine serum for 1 h at 37°C, washed three times with PBS, incubated with the FITC-labeled Rabbit anti-bovine antibody at 37°C for 1 h, washed three times with PBS once more, and finally incubated with the Alexa Fluor 555-anti-His antibody at 37°C for 1 h. Nuclei were counterstained with DAPI (blue). Cell samples were examined with a fluorescence microscope (Leica EL 6000). The karyomorphism of MDBK cells was observed under a CLSM (Leica).

Since BoScFv-PE38 had the ability to bind to MDBK cells infected with BoHV-1, it was important to determine whether BoScFv-PE38 immunotoxin had selective cytotoxic effect on cells infected with BoHV-1. Initially, we estimated the cytotoxic concentration of BoScFv-PE38 immunotoxin required to induce specific cytotoxic effect on BoHV-1 infected MDBK cells. The MTS assays showed a reduced cell viability when BoScFv-PE38 immunotoxin concentration was increased to 1.25 μM in culture medium, indicating that the cytotoxic concentration of BoScFv-PE38 immunotoxin for MDBK cells should not exceed to 1.25 μM and the cytotoxic concentration of 50% (CC_50_) was 2.25 μM (**Figure 5A**). The cell proliferation assays clearly demonstrated that a dose of 1.00 μM BoScFv-PE38 immunotoxin can significantly inhibit (*P* < 0.001) the proliferation the MDBK cells infected with range of 0.05–2.5 multiplicities of infection (MOIs) of BoHV-1(**Figure 5B**). Based on these results, we infected MDBK cells with 1.0 MOI of BoHV-1 and then treated the cells with gradient concentrations of BoScFv-PE38 immunotoxin. The data revealed that the proliferation of MDBK cells was continually reduced with the increase of BoScFv-PE38 immunotoxin concentration (**Figure 5C**). Suggesting that the BoScFv-PE38 immunotoxin exerted dose-dependent cytotoxic effects on BoHV-1-infected MDBK cells. To confirm the above findings, we observed the karyomorphism of MDBK cells infected with BoHV-1 after being treated with BoScFv-PE38 immunotoxin. Confocal microscopy images corroborated above findings indicating the nuclei of BoHV-1-infected MDBK cells were defective after treatment with 1.00 μM BoScFv-PE38, whereas untreated cells maintained normal morphology until 18 h post-infection despite infection with equal infectious dose of BoHV-1 (**Figure 5D**).

### BoScFv-PE38 Immunotoxin Induces Apoptosis of BoHV-1-Infected Cells

**FIGURE 6:**
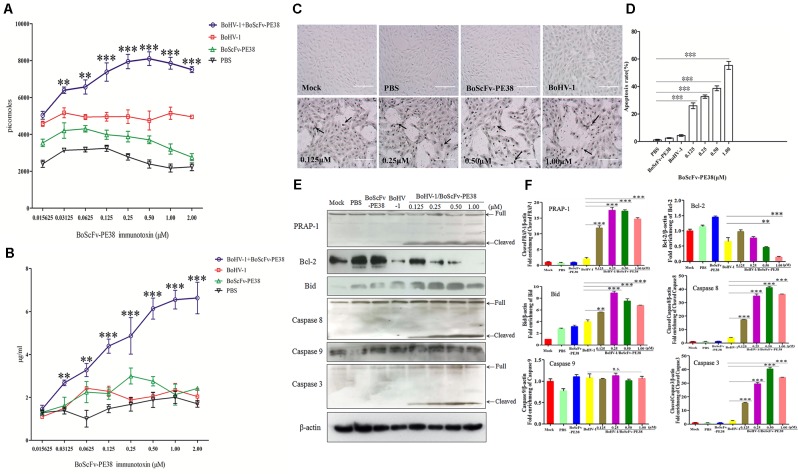
Apoptosis of BoHV-1-infected MDBK cells was induced by BoScFv-PE38. **(A)** The titration of ATP in BoHV-1-infected MDBK cells treated with BoScFv-PE38. MDBK cells were seeded in 96-well plates and cultured to 90% confluency at 37°C for 18–24 h. Then, the MDBK cells were infected with BoHV-1 (MOI = 1), added to the culture medium, and cultivated 37°C for 1.5 h. The culture medium was subsequently replaced with new culture medium containing 1% FBS and BoScFv-PE38 (0.015625, 0.03125, 0.0625, 0.125, 0.25, 0.50, 1.00, and 2.00 μM), and the MDBK cells were cultured for 24 h. The concentration of ATP was measured with a kit according to the manufacturer’s instructions. **(B)** The titration of ammonia in BoHV-1-infected MDBK cells treated with BoScFv-PE38. MDBK cells were treated as described above, and the concentration of ammonia was measured with a kit. Statistical analyses were performed via analysis of variance (ANOVA) using SPSS software, version 18.0, and the fitting curves of various concentrations of BoScFv-PE38 were drawn with GraphPad Prism 5.0. “^∗∗^” represents statistically significant differences (*P* < 0.01). “^∗∗∗^” represents statistically significant differences (*P* < 0.001). **(C)** The TUNEL assay. MDBK cells were seeded in 24-well plates and cultured to 90% confluency at 37°C for 18–24 h. Then, the MDBK cells were infected with BoHV-1 (MOI = 1), added to the culture medium, and cultivated at 37°C for 1.5 h. The culture medium was subsequently replaced with new culture medium containing 1% FBS and BoScFv-PE38 (0.125, 0.25, 0.5, and 1 μM), and the MDBK cells were cultured for 24 h. The apoptosis-positive MDBK cells were detected with the *In Situ* Cell Death Detection Kit, AP (Roche) according to the manufacturer’s instructions. The arrows indicate the TUNEL-positive apoptotic cells. **(D)** Apoptotic rates of BoHV-1-infected MDBK cells in the presence of different concentrations of BoScFv-PE38. The TUNEL-positive MDBK cells were counted, and statistical analyses were performed via analysis of variance (ANOVA) using SPSS software, version 18.0. The fitting curves of various concentrations of BoScFv-PE38 were drawn with GraphPad Prism 5.0. “^∗∗∗^” represents statistically significant differences (*P* < 0.001). **(E)** Expression of apoptosis proteins in BoHV-1-infected MDBK cells after treatment with BoScFv-PE38. MDBK cells were seeded in six-well plates and cultured to 90% confluency at 37°C over 18–24 h. Then, the MDBK cells were infected with BoHV-1 (MOI = 1), added to the culture medium, and cultivated at 37°C for 1.5 h. The culture medium was subsequently replaced with new medium containing 1% FBS and BoScFv-PE38 (0.125, 0.25, 0.5, and 1 μM), and the MDBK cells were cultured for an additional 24 h. The whole-cell proteins were separated via SDS-PAGE and transferred to PVDF membranes. The membranes were subsequently hybridized with antibodies against PARP-1, Bcl-2, Bid, caspase-8, caspase-8 and β-actin, and the blots were developed using Super Signal chemoluminescent substrates. **(F)** Apoptosis proteins quantification of band intensity by Image J software, representative results are displayed with graphs corresponding to the ratios of cleaved PRAP-1, Bcl-2, Bid, caspase 8, caspase 9 and caspase 3 normalized to theβ-actin control conditions. The data were analyzed using SPSS software, and the graph was produced using GraphPad Prism 5.0. “n.s.” represents no statistically significant differences (*P* > 0.05). “^∗∗^” represents statistically significant differences (*P* < 0.01). “^∗∗∗^” represents statistically significant differences (*P* < 0.001).

To explore the mechanism of the specific cytotoxic effect of BoScFv-PE38 immunotoxin on the BoHV-1 infected MDBK cells, we analyzed the concentrations of adenosine triphosphate (ATP) and ammonia, which are the major indicators associated with cytotoxicity, in BoScFv-PE38 treated, BoHV-1-infected MDBK cells (Leist et al., 1997; Cheng et al., 2015). The results showed that the concentrations of ATP and ammonia in BoHV-1-infected MDBK cells treated with BoScFv-PE38 were significantly higher than in cells that were infected with BoHV-1 alone, non-infected cells treated with BoScFv-PE38, and negative cells (mock treated with PBS). Additionally, the titers of ATP and ammonia increased with the concentration of BoScFv-PE38 within 0.015625–1 μM or 0.015625–2.00 μM, respectively (**Figures 6A,B**). This finding demonstrated that BoScFv-PE38 immunotoxin increased the production of ATP and ammonia in BoHV-1-infected MDBK cells. Observed high levels of ATP or ammonia imply that cell death was induced by apoptosis. The apoptotic cells were further subjected to terminal deoxynucleotidyl transferase dUTP nick end labeling (TUNEL) assays. Increased amount of apoptotic bodies were observed in BoHV-1-infected and BoScFv-PE38 immunotoxin treated MDBK cells compared with mock treated BoHV-1 infected cells (**Figure 6C**). The rate of apoptosis induced by BoScFv-PE38 immunotoxin treated BoHV-1infected cells was significantly higher than in the mock PBS-treated control or following induction by BoScFv-PE38 immunotoxin or BoHV-1 individually. Additionally, there was a dose-dependent increase in the rate of apoptotic cells induced by BoScFv-PE38 immunotoxin bound with BoHV-1 (**Figure 6D**). These results were further supported by the analysis of apoptotic proteins including PRAP-1, Bcl-2, Bid, Caspase 8 and Caspase 3. The results clearly revealed increased cleavage of pro-apoptotic proteins Bid in BoHV-1-infected MDBK cells after treatment with BoScFv-PE38 immunotoxin, while the expression of the anti-apoptosis protein Bcl-2 was relatively decreased (**Figures 6E,F**).

### BoScFv-PE38 Effectively Inhibits the Infectivity of BoHV-1 in MDBK Cells

**FIGURE 7:**
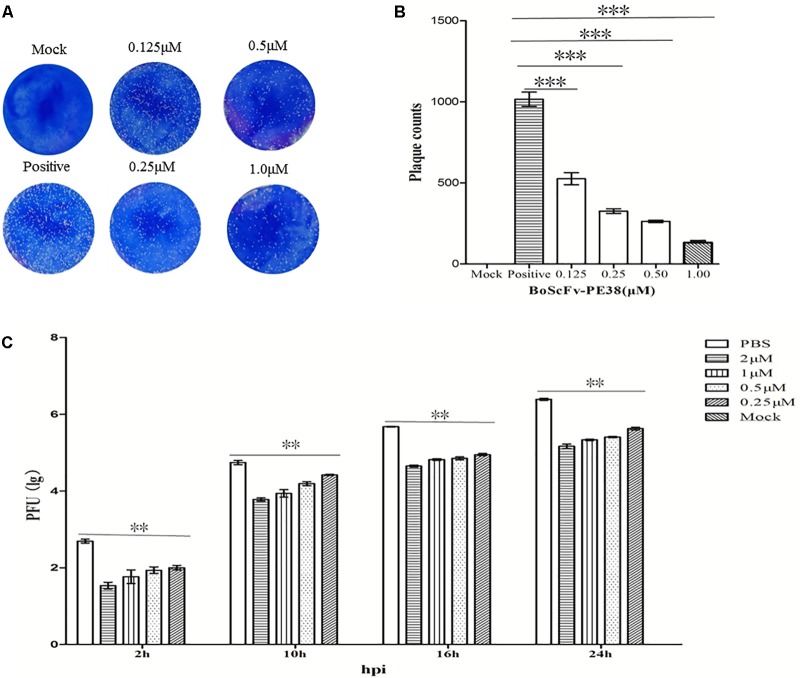
The infectivity of BoHV-1 was inhibited by BoScFv-PE38. **(A,B)** Plaque reduction neutralization test of BoScFv-PE38. Mock represents normal MDBK cells; positive represents MDBK cells infected with BoHV-1 and treated with PBS. MDBK cells were infected with BoHV-1 and treated with different concentrations of BoScFv-PE38 (0.125, 0.25, 0.5, and 1.0 μM). Viral plaques were counted using 0.1% toluidine blue in saline. The data were analyzed using SPSS software, and the graph was produced using GraphPad Prism 5.0. “^∗∗∗^” represents statistically significant differences (*P* < 0.001). **(C)** BoScFv-PE38 antiviral activity in ICAs. MDBK cells were seeded in six-well plates and cultured to 90% confluency at 37°C over 18–24 h. Then, the MDBK cells were infected with BoHV-1 (MOI = 1), added to the culture medium, and cultivated at 37°C for 1.5 h. The culture medium was subsequently replaced with new medium containing 1% FBS and BoScFv-PE38 (0.125, 0.25, 0.5, and 1 μM), and the MDBK cells were cultured for an additional 24 h. The whole culture was harvested and repetitively freeze-thawed three times, at 2, 10, 16, and 24 h. Then, virus titers were measured in plaque forming units (PFU), and viral plaques were counted visually. The data were analyzed using SPSS software. “^∗∗^” represents statistically significant differences (*P* < 0.01).

The observed data demonstrated that BoScFv-PE38 immunotoxin specifically targets BoHV-1-infected cells and exerts cytotoxic effects. Therefore, we examined the ability of BoScFv-PE38 immunotoxin to inhibit the infectivity of BoHV-1 in MDBK cells using micro-neutralization tests and plaque reduction assays. As shown in **Figure 7A**, the BoHV-1 plaque count in MDBK cells was significantly reduced (*P* < 0.01) following pre-incubation of virus with varying concentrations of BoScFv-PE38 immunotoxin for 1 h compared with mock PBS treatment (**Figure 7B**). The inhibitory effect of BoScFv-PE38 immunotoxin on BoHV-1 replication was also evaluated via the infectious center assay (ICA). The results showed that there were a marked decrease in BoHV-1 virus replication in MDBK cell cultures supplemented with a varying of amount (1, 0.5, 0.25, and 0.125 μM) of BoScFv-PE38 immunotoxin (**Figure 7C**). The estimated 50% inhibitory concentration (IC_50_) of BoScFv-PE38 immunotoxin was 4.95 ± 0.33 nM observed within 24 h of infection using viral plaque reduction assays. These results illustrated that BoScFv-PE38 effectively inhibited the infectivity of BoHV-1 in MDBK cells. From these results, selective index (SI) can be inferred to be 456 ± 31 in terms of the IC_50_ value of the cytotoxic concentration (Supplementary Table S2).

## Discussion

Immunotoxins are novel therapeutic agents that have previously been developed for use against human viruses and cancer. Currently, there is dearth of research into the development of immunotoxins for use against viruses of farmed animals, which have significant economic impacts worldwide (Liu et al., 2012). But it is necessary to develop new therapeutics protects those valuable cattle from infectious diseases; in particular the viral diseases to overcome limitations of vaccination approaches (Levings and Roth, 2013). Since recombinant immunotoxins represent a kind of therapeutics consisting of a cytotoxic agent fused to a variable antibody fragment; these agents bind specifically to target cells and exert cytotoxic effects (Berger and Pastan, 2010; Margolis et al., 2016), we therefore developed a novel recombinant BoScFv-PE38 immunotoxin that target BoHV-1 infected cells and block virus replication and decimation. The recombinant BoScFv-PE38 immunotoxin was generated by fusing the scFv fragment of an anti-BoHV-1 gD protein monoclonal antibody with the bacterial toxin PE38. To eliminate the non-specific cytotoxicity of normal cells, the natural binding domains of bacterial toxins are truncated and connected with a flexible 16-aa linker peptide (GGGGS)_3_, which has been reported as the best linker peptide (Chatterjee et al., 2012; Schotte et al., 2014). In this fusion protein, PE38 lacks its natural PE-binding domain to avoid an effect on non-infected cells, and scFv only target to the gD protein of BoHV-1 and exert its effect by killing and eradicating the pool of infected cells (**Figures 1A–C**). The results of SDS-PAGE analysis demonstrated that the recombinant protein was the desired immunotoxin, BoScFv-PE38 (**Figures 2A,B**).

Immunotoxins must exhibit high efficacy and specificity for binding to target viral antigens, together with inducing minimal side effects or toxicity to the non-infected cells, to be effective therapeutic agents (Brinkmann et al., 1993; Hanke et al., 2016; Spiess et al., 2016). To evaluate the ability of the BoScFv-PE38 immunotoxin to specifically bind to BoHV-1 envelope protein gD, the purified BoScFv-PE38 protein was labeled with horseradish peroxidase (HRP) and analyzed via western blotting and ELISA. Western blot analysis showed that the BoScFv-PE38 protein could specifically react with either the recombinant gD protein or BoHV-1 virus (**Figures 2C,D**). Furthermore, the BoScFv-PE38 immunotoxin bound BoHV-1 and gD with dissociation constants (Kd) of 97.63 nM and 12.81 nM, respectively (**Figures 2E,F**). The ability of BoScFv-PE38 immunotoxin to capture BoHV-1 was also demonstrated by a double sandwich ELISA (**Figure 2G**). These results indicated that the antibody portion of the recombinant immunotoxin possessed high affinity and specificity against the BoHV-1 virus. Since gD is the major antigen that induces neutralizing antibodies and is involved in virus penetration during BoHV-1 infection, gD has been considered the major target for antiviral agents and vaccines (Alves et al., 2014; Kumar et al., 2014). Therefore, it is expected that the gD protein is targeted by BoScFv-PE38 immunotoxin, not only because it is present in the viral envelope but also because it is expressed abundantly on the surface of BoHV-1-infected cells (Liu et al., 2017; Müller et al., 2017). To confirm the binding specificity of BoScFv-PE38 immunotoxin, the gD protein was expressed in 293T cells and detected via IFA using BoScFv-PE38 as a primary antibody. Confocal imagining revealed that the location of the gD protein detected by BoScFv-PE38 was similar to that indicated by its GFP fusion protein, suggesting that the cells bound by BoScFv-PE38 immunotoxin were expressing gD (**Figures 3A,B**). Thus, the binding affinity of BoScFv-PE38 immunotoxin to gD is highly specific. We further evaluated the cytotoxicity of BoScFv-PE38 immunotoxin to 293T cells expressing gD. The results showed that BoScFv-PE38 immunotoxin could significantly reduce the proliferation of 293-T cells expressing gD through a dose-dependent manner, but there was no obvious deleterious effect to the untreated control cells (**Figures 3C,D**), suggesting that BoScFv-PE38 immunotoxin produced cytotoxic effects only to the cells expressing gD protein and negligible effect on uninfected cells. Since BoScFv-PE38 immunotoxin is composed of a targeting molecule scFv, the ability to recognize its corresponding antigen should be an inherent property (Ledford, 2011). In this study, we found that BoScFv-PE38 immunotoxin could specifically recognize BoHV-1 infected MDBK cells (**Figures 4A,B**). As an anti-BoHV-l therapeutics, it is necessary to eradicate viral infections. Thus, the immunotoxin must also have a capability to rapidly internalize the infected cells (Spiess et al., 2016). Internalization of BoScFv-PE38 immunotoxin was verified in this study, as it was found in the cytoplasm of MDBK cells infected with BoHV-1 (**Figure 4C**), which implied that the toxin PE38 can be efficiently delivered to the intracellular environment and play a crucial role in eliminating BoHV-1 (Frizzo da Silva et al., 2013). Confocal microscopy images provided further evidence that the nucleus of MDBK cells infected with BoHV-1 was damaged after treatment with 1.00 μM BoScFv-PE38 (**Figure 5D**). These findings clearly indicated the internalization capability of BoScFv-PE38 via entrapment of BoHV-1 after binding to gD, which is similar to the antitumor immunotoxin 4D5scFv-PE40 reported earlier (Sokolova et al., 2017).

The assessment of cytotoxic activity of BoScFv-PE38 immunotoxin via the MTS assay also revealed a minimal residual endotoxin effect on uninfected cells with study cytotoxic concentration up to 1.25 μM and the CC_50_ was 2.25 μM (**Figure 5A**). Next, the BoScFv-PE38 immunotoxin showed signification inhibition of BoHV-1 replication and exerted cytotoxic effects to the virus infected cells (**Figures 5B,C**). Previous studies have shown that cytotoxicity to targeted cells induces apoptosis in virus-infected host cells or cancer cells (Liu X.F. et al., 2016; Sokolova et al., 2017); specifically, high levels of ATP and ammonia are the major inducers of apoptosis (Jin et al., 2017; Zhang et al., 2017). In our study, BoScFv-PE38 significantly increased the titers of ATP and ammonia in MDBK cells infected with BoHV-1 (**Figures 6A,B**), which implied that apoptosis was triggered in MDBK cells as a result of treatment with BoScFv-PE38 immunotoxin and the effect was dose-dependent (**Figures 6C,D**). The analysis of pro and anti-apoptotic proteins including PRAP-1, Bcl-2, Bid, Caspase 8 and Caspase 3 also corroborated the specific apoptotic activity of BoScFv-PE38 leading to cytotoxic effect on BoHV-1 infected cells (**Figures 6E,F**).

Importantly, our results demonstrated that BoHV-1 virus production was significantly reduced in MDBK cells by treatment with BoScFv-PE38 immunotoxin (**Figures 7A–C**), whereas the IC_50_ value was 4.95 ± 0.33 nM; therefore, the SI was calculated as 456 ± 31 in terms of cytotoxic concentration (Supplementary Table S2). The results clearly demonstrated the inhibitory activity of BoScFv-PE38 immunotoxin for BoHV-1. Considering the neutralization activity of scFv in our previous study (Xu et al., 2017), the use of antibody portion scFv within BoScFv-PE38 immunotoxin could also target cell free virions during cytolytic phase of BoHV-1 infection in cattle. We therefore, conclude that our developed BoScFv-PE38 immunotoxin had an ability to target BoHV-1 at lytic phases of infection. Taken together, the findings of this study demonstrate that this recombinant immunotoxin could be a potential therapeutic agent for controlling and treating viral pathogens affecting at animals.

## Author Contributions

YL and JX: conceived and designed the experiments. JX, XL, BJ, XF, JW, YC, and XZ: performed the experiments. JX and YL: analyzed the data. XH: contributed reagents/ materials/analysis tools. JX and YL: wrote the paper. MI and JS: modified the paper.

## Conflict of Interest Statement

The authors declare that the research was conducted in the absence of any commercial or financial relationships that could be construed as a potential conflict of interest. The reviewer BFM and handling Editor declared their shared affiliation.

## Footnotes

1 https://www.ncbi.nlm.nih.gov/protein/553773623

2 http://www.expasy.ch/tools/scanprosite/
