# Supplementary material for: Antiviral Immunotoxin Against Bovine herpesvirus-1: Targeted Inhibition of Viral Replication and Apoptosis of Infected Cell
Source: Front Microbiol. 2018 Apr 4;9:653. doi: 10.3389/fmicb.2018.00653 (PMC5893756; doi:10.3389/fmicb.2018.00653)
Supplement: Supplementary file 1 [file Presentation_1.PDF]

# Antiviral immunotoxin against *Bovine herpesvirus*-1: targeted inhibition of viral replication and apoptosis of infected cell

***Jian Xu[1,#], Xiaoyang Li[1,2,#], Bo Jiang[1,#], Xiaoyu Feng[3], Jing Wu[1,2], Yunhong Cai[1], Xixi Zhang[1], Xiufen Huang[1], Joshua E. Sealy[4], Munir Iqbal[4] and Yongqing Li[1]**** 

*[1] Institute of Animal Husbandry and Veterinary Medicine, Beijing Academy of agricultural and Forestry Sciences, Beijing, P. R. China, 100097. [2] College of Animal Science and Technology, Jiangxi Agricultural University, Nanchang, Jiangxi, P. R. China, 330045. [3] Beijing center for animal disease control and prevention, Beijing, P. R. China, 100260. [4] The Pirbright Institute, Ash Rd, Pirbright, Woking, GU24 0NF, United Kingdom*

***Correspondence:** Yongqing Li, email**:** liyongqing@iasbaafs.net.cn*

*[#]These authors contributed equally to this work.*

*Tel.: +86-10-51503195 Fax: +86-10-51503195*

**Fig. S1. Immunofluorescence assay analysis of BoHV-1 gD expressed in 293T cells..**

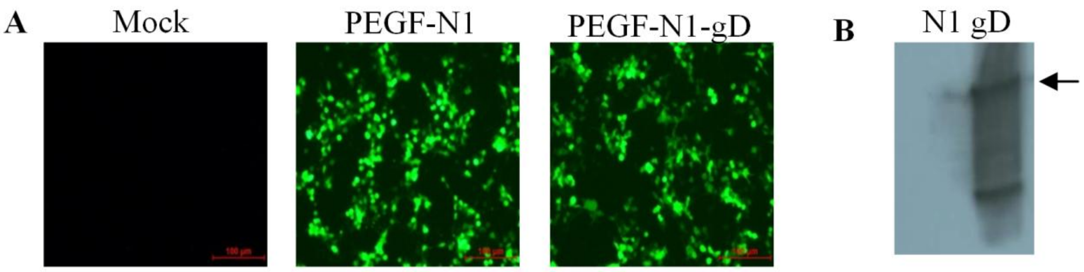


**A:** The green fluorescent image of BoHV-1 gD expressed in 293T cells. 293T cells were seeded into six-well (or 96-well) plates before the experiment, and when the cells reached 70% confluence, the PEGF-N1 and PEGF-N1-gD plasmids were transfected with Lipofectamine 3000 (Life Technology, USA) according to the manufacturer's instructions. The 293T cells were subsequently cultured for an additional 18-24 h. Cell samples were examined with a fluorescence microscope (Leica EL 6000).

B: The expression of BoHV-1 gD in the 293T cells was detected by western blotting. 293T cells transfected with the PEGF-N1 and PEGF-N1-gD plasmids were cultured for 18-24 h and then harvested for separation via SDS-PAGE, followed by transfer to PVDF membranes. The proteins were subsequently hybridized with the McAb–gD antibody, and the blots were developed using Super Signal chemoluminescent substrates.

**Table S1. Amino acid sequence of BoScFv-PE38 used in this study.**

| Protein domain | Sequences | Position |
|---|---|---|
| BoHV-1 ScFv<br>(Bind domain ) | MAEEVKLQQSGGGLVQPGESLKLSCESNECEFPSYNISWVRKTPGKSLDLVAAIKSGYYVD<br>TMERRFIISRDNTKKTLYLQMSSLRSEDAALYYCARRGIITTIGAKGPRSPSPQGGGGSGGG<br>GSGGGGSDIVMTQTPLSLSVSLGDQASISCRSSQSIVHSNGNTYFEWYLQKPGQSPRLLIYK<br>VSNRFSGVPDRFSGSGSGTDFTLKISRVEAEDLGVYYCSQGSLIPFTFGSGTKLEIKR | 1-243AA |
| 3($G_4$S) Linker | GGGGSGGGGSGGGGS | 244-258AA |
| PE38<br>(Cytotoxic domain) | PEGGSLAALTAHQACHLPLETFTRHRQPRGWEQLEQCGYPVQRLVALYLAARLSWNQVD<br>QVIRNALASPGSGGDLGEAIREQPEQARLALTLAAAESERFVRQGTGNDEAGAASADVVS<br>LTCPVAAGECAGPADSGDALLERNYPTGAEFLGDGGDISFSTRGTQNWTVERLLQAHRQL<br>EERGYVFVGYHGTFLEAAQSIVFGGVRARSQDLDAIWRGFYIAGDPALAYGYAQDQEPDA<br>RGRIRNGALLRVYVPRSSLPGFYRTGLTLAAPEAAGEVERLIGHPLPLRLDAITGPEEEGGR<br>LETILGWPLAERTVVIPSAIPTDPRNVGGDLDPSSIPDKEQAISALPDYASQPGKPPREDLK | 259-621AA |

**Table S2. 50% inhibitory concentration (IC50) and selective index (SI) values of BoScFv-PE38 in BoHV-1-infected MDBK cells.**

| BoScFv-PE38(nM) | Rate of CPE | Number of CPE | Number of survival | Total | | | | Repeated | $IC_{50}$(nM) | SI value |
|---|---|---|---|---|---|---|---|---|---|---|
| | | | | Number of CPE | Number of survival | Rate of CPE | Rate of Survival (%) | | | |
| 125 | 0/8 | 0 | 8 | 0 | 23 | 0/23 | 100 | Trial I | 4.98 | 452 |
| 31.25 | 2/8 | 2 | 6 | 2 | 15 | 2/17 | 88 | | | |
| 7.81 | 3/8 | 3 | 5 | 5 | 8 | 5/13 | 62 | | | |
| 1.95 | 5/8 | 5 | 3 | 10 | 4 | 10/14 | 29 | | | |
| 0.49 | 7/8 | 7 | 1 | 17 | 1 | 17/18 | 5 | | | |
| 0.12 | 8/8 | 8 | 0 | 25 | 0 | 25/25 | 0 | | | |
| 125 | 0/8 | 0 | 8 | 0 | 23 | 0/23 | 100 | Trial II | 4.54 | 496 |
| 31.25 | 2/8 | 1 | 7 | 2 | 15 | 2/17 | 88 | | | |
| 7.81 | 3/8 | 3 | 5 | 4 | 8 | 4/12 | 67 | | | |
| 1.95 | 5/8 | 5 | 3 | 9 | 3 | 9/12 | 25 | | | |
| 0.49 | 8/8 | 8 | 0 | 17 | 0 | 17/17 | 0 | | | |
| 0.12 | 8/8 | 8 | 0 | 25 | 0 | 25/25 | 0 | | | |
| 125 | 0/8 | 0 | 8 | 0 | 22 | 0/22 | 100 | Trial III | 5.34 | 421 |
| 31.25 | 2/8 | 2 | 6 | 2 | 14 | 2/16 | 87 | | | |
| 7.81 | 3/8 | 3 | 5 | 5 | 8 | 5/13 | 61 | | | |
| 1.95 | 5/8 | 5 | 3 | 10 | 3 | 10/13 | 23 | | | |
| 0.49 | 8/8 | 8 | 0 | 18 | 0 | 18/18 | 0 | | | |
| 0.12 | 8/8 | 8 | 0 | 26 | 0 | 26/26 | 0 | | | |
